# Selective targeting of the TLR4 co-receptor, MD2, prevents colon cancer growth and lung metastasis

**DOI:** 10.7150/ijbs.39098

**Published:** 2020-02-17

**Authors:** Vinothkumar Rajamanickam, Tao Yan, Shanmei Xu, Junguo Hui, Xiaohong Xu, Luqing Ren, Zhoudi Liu, Guang Liang, Ouchen Wang, Yi Wang

**Affiliations:** 1 Chemical Biology Research Center, School of Pharmaceutical Sciences, Wenzhou Medical University, Wenzhou, Zhejiang, 325035, P. R. China.; 2 Department of Surgery, the First Affiliated Hospital of Wenzhou Medical University, Wenzhou, Zhejiang, 325035, P. R. China.

**Keywords:** colon cancer, MD2, TLR4, NF-κB, colitis-induced colon cancer

## Abstract

Toll-like receptor (TLR) signaling is an emerging pathway in tumor cell invasion and metastasis. Myeloid differentiation protein-2 (MD2) contributes to ligand recognition and activation of TLRs in response to exogenous microbial insults or endogenous agents. We hypothesized that blocking MD2 using a specific inhibitor would prevent TLR4-mediated inflammatory responses and metastatic cancer growth. Here, we report that a MD2 inhibitor, L6H21, inhibited migration and invasion of LPS-activated colon cancer CT26.WT cells. These activities were accompanied by inhibition of nuclear factor-κB (NF-κB) activation, and thereby inhibition of the production of pro-inflammatory cytokines and adhesive molecules in colon cancer cells. Furthermore, L6H21 inhibited CT26.WT metastasis to the lung in BALB/c mice as well as suppressed colitis-induced colon cancer induced by azoxymethane/dextran sulfate sodium (AOM/DSS). Taken together, our results demonstrated that L6H21 suppressed tumor invasion and metastasis through blocking TLR4-MD2/NF-κB signaling axis. These findings reveal that inhibition of MD2 may be an important target for the development of colon cancer therapies.

## Introduction

Colorectal cancer (CRC) is the third most commonly diagnosed cancer in both men and women, and the fourth leading cause of cancer-related deaths worldwide. Recently, the incidence of CRC is rapidly increasing in many Asian countries, including China, Japan, Korea and Singapore, bringing the urgent need to discover effective therapy for the treatment of CRC 1, 2. Initiation of CRC is multifactorial and includes environmental factors, diet, genetic predisposition and epigenetic alterations in the colonic epithelium. There is accumulating evidence which indicates chronic inflammation as an important factor in the development of CRC 3, 4. The toll-like receptor (TLR) family constitutes a critical part of the innate immune system 5, which has been shown to be important for maintenance of intestinal epithelial homoeostasis and regulation of tissue repair in intestinal epithelial cells 6. In particular, TLR4, the receptor for bacterial lipopolysaccharide (LPS), is implicated in the development of CRC. For example, elevated TLR4 expression has been found in CRC 7. Furthermore, increased expression of TLR4 correlates with upregulation inflammatory cytokines and higher possibility of cancer recurrence in CRC patients 8. In addition, TLR4 signaling is associated with colitis-associated neoplasia and CRC metastasis 9. Based on the strong findings linking TLR4 and CRC, targeting TLR4 signaling pathway is attractive for the treatment of CRC.

Myeloid differentiation protein 2 (MD2), the co-receptor for TLR4, is required for LPS recognition and the subsequent TLR4 activation 10. After binding to LPS, TLR4-MD2-LPS complex activates the downstream inflammatory cascades through myeloid differentiation primary-response gene 88 (MyD88) and the Toll/IL-1R domain-containing adaptor-inducing interferon β (TRIF) 11, suggesting that targeting MD2 to block the binding of LPS to TLR4 could be a potential pharmacological strategy for CRC treatment. Actually, Virginie et al. has reported that overexpression of MD2 promotes colon cancer proliferation and migration via activating EGFR pathway *in vitro*. Zou et al. reported that sTLR4/MD2 complex significantly attenuates LPS induced pro-inflammatory and migration-related cytokine production, and protects mice from tumor both in xenograft and implantation metastasis model 12. However, how MD2 involves in the development of CRC *in vivo* is still unclear. In this study, we utilized a specific MD2 inhibitor, L6H21 13, and *MD2^-/-^* mice to evaluate the role of MD2 in CRC tumorigenesis and metastasis.

## Materials and Methods

### Chemicals and reagents

Dulbecco's Modified Eagle's Medium (DMEM), RPMI-1640 media, and heat-inactivated fetal bovine serum (FBS) were obtained from Gibco/BRL life Technologies (Eggenstein, Germany). Cell culture penicillin-streptomycin supplement was purchased from Mediatech Inc. (Manassas, VA). Antibodies against MD2, VCAM-1 and p-IκBα were purchased from Abcam (Cambridge, MA). Antibodies against NF-κB p-p65 was purchased from Cell Signaling (Danvers, MA, USA). Antibodies against TLR4, IκBα, NF-κB p65 subunit, MMP2, MMP9, ICAM-1, GAPDH, goat anti-rabbit IgG-HRP, mouse anti-goat IgG-HRP and donkey anti-goat IgG-HRP were obtained from Santa Cruz Biotechnology (Santa Cruz, CA). Anti-MD2 neutralizing antibody was obtained from Thermo Fisher (Waltham, MA). Matrigel was purchased from BD Biosciences (Shanghai, China). Mitomycin C was purchased from Sigma-Aldrich (Louis, MO).

### L6H21 synthesis

The compound of interest, chalcone derivative L6H21, was synthesized and characterized in our laboratory as described previously 14. The compound, with a purity of 98.9%, was dissolved in DMSO for *in vitro* experiments and in 1% CMC-Na for *in vivo* experiments.

### Cell lines and maintenance

Cell lines were obtained from Shanghai Institute of Biosciences and Cell Resources Center (Chinese Academy of Sciences, Shanghai, China). We utilized human colon cancer cells, SW620 (RRID: CVCL_0547) and HCT116 (RRID: CVCL_0291), and mouse colon cancer cells, CT26.WT (CVCL_7256), for these studies. Normal human embryonic kidney cells, HEK-293 (RRID: CVCL_0045), were used as control to assess MD2 expression. Human colon cancer cells were grown in DMEM and mouse cells in RPMI-1640 medium. Both formulations were supplemented with 10% heat-inactivated FBS and 1% penicillin-streptomycin.

### Human Subjects

The study was approved by the Human Ethical Committee of the First Affiliated Hospital of Wenzhou Medical University (Approval document #2014-35), and informed consent was obtained from the patients. Donors of colon cancer tissue were obtained from patients admitted for CRC surgery at the First Affiliated Hospital of Wenzhou Medical University. The age of male donors was 39-86 years (n=34), and of female donors 35-84 (n=16). Tumor tissue and adjacent tissue were collected for histological examination.

### Experimental animals

Animal care and experimental protocols were approved by the Committee on Animal Care of Wenzhou Medical University (Wenzhou, Zhejiang, China; Approval document wydw2014-0062), and all animals received humane care according to the National Institutes of Health (USA) guidelines. Male BALB/c mice weighing 18-20 g (7-8 weeks old) were obtained from the Beijing Vital River Laboratory Technology Co. (Beijing, China). Male C57BL/6 mice (7-8 weeks old) were obtained from Model Animal Resource Information Platform (Nanjing, China). Male MD2^-/-^ mice (B6.129P2-Ly96<tmlKmiy>) with a C57BL/6 background were provided by RIKEN BioResource Center of Japan (Tsukuba, Ibaraki, Japan). Animals were housed in a standard vivarium with 12:12 hour light-dark cycle, 25±2°C temperature, and relative humidity of 50±10%. Mice were fed a standard rodent diet and given water *ad libitum*. The animals were acclimatized to the laboratory for at least 7 days before initiation of the study. The results of all studies involving animals were reported in accordance with these guidelines and protocols.

### Induction of CT26.WT metastasis mouse model

Cultured CT26.WT cells were harvested and injected intravenously (i.v.) into the tail vein of BALB/c mice at 3×10^5^ cells/mouse in 200 μL of RPMI-1640. Mice were administered L6H21 (30 or 60 mg/kg orally) or vehicle (1% CMC-Na) and evaluated for up to 60 days. Control mice (without CT26.WT) were treated with L6H21 at 60 mg/kg and served as the drug control (n=10 in each group). The survival and body weight of the mice were recorded weekly.

A parallel set of experiments was made to monitor tumor nodule establishment in the lungs of mice within the survival period. After the treatment protocol, mice were sacrificed at 22^nd^ day for analysis.

### Induction of colitis-induced colon cancer

In this colitis cancer model, mice were treated with the genotoxic agent azoxymethane (AOM), followed by dextran sulfate sodium (DSS) in drinking water to induce chronic inflammation. Forty WT and 20 MD2^-/-^ mice were randomized into 6 experimental groups: control mice (n = 8, WT, no treatment), L6H21-treated WT mice (n = 8, L6H21, treated orally, 60 mg/kg daily), AOM/DSS (n = 12, WT), AOM/DSS treated with L6H21 (n = 12, WT, L6H21, treated orally, 60 mg/kg daily), AOM/DSS in MD2^-/-^ (n = 12, MD2^-/-^ mice), and MD2^-/-^ control mice (n = 8, MD2^-/-^, no AOM/DSS). AOM was administered at 10 mg/kg in PBS by intraperitoneal (i.p.) injection on the first day of the experiment. Then, mice received 3 cycles of 2.5% DSS through drinking water every day on weeks 2, 4, and 6. The experiment was terminated at the end of week 20, with the survival rate and body weights monitored throughout the experimental period. Colon tumor specimens were harvested for qPCR analysis, Western blot analysis, IL-6 determination by ELISA, and histological evaluation. Fecal occult blood test was also performed to detect for hemorrhage from gastrointestinal tract.

### Cell viability assay

Mouse CT26.WT and human SW620 and HCT116 cells were seeded in respective growth medium at a density of 5×10^3^ cells/well in 96-well plates. Cells were allowed to attach for 24 h, and treated with increasing concentrations of L6H21 for 1 h. In addition, viability was measured following MD2 knockdown by siRNA or functional neutralization by anti-MD2 antibody (1 μg/mL; 1 h). Following pretreatment, cells were challenged with 1 μg/mL LPS for indicated time points. At the end of the treatments, freshly prepared MTT reagent was added, and cells were incubated for 4 h. Formazan crystals resulting from mitochondrial enzymatic activity were dissolved with DMSO and absorbance was determined at 490 nm. Each experiment was done in triplicates and repeated three times. Results were expressed as percent of vehicle control. IC_50_ values of each compound were calculated by GraphPad Pro 5.0 (San Diego, CA).

### Matrigel invasion assay

The effects of MD2 blockade on invasion of CT26.WT cells were studied in Matrigel-coated Transwell plates with 8 μm pore size filters (Corning Costar Corp, Shanghai, China). The filters were coated with 10 μL of Matrigel (BD Biosciences) diluted 1:9 in serum-free medium. Cells at 5×10^5^ density were suspended in 200 μL serum-free RPMI 1640 and plated onto the filter of the upper chamber. Following attachment, cells were exposed to L6H21 (10 μM), MD2 neutralizing antibody (1 μg/mL), or DMSO used as vehicle control for 1 h. Similarly, MD2 siRNA-transfected cells were also plated in Transwells for study. Each experimental cell group was challenged with 1 μg/mL LPS. The bottom chamber was loaded with 500 μL of RPMI 1640 medium containing 10% FBS. After 24 h, the cells were fixed in 1% methanol for 15 min and stained with crystal violet for 25 min. Images of cells that have migrated through the Matrigel to the underside of the filter were captured using Nikon microscope equipped with a digital camera (Nikon, Japan). Total number of invading cells was counted and presented as the ratio to DMSO control.

### Scratch/ wound model

Cells at a density of 5×10^5^ cells/well were seeded in 6-well plates and allowed to adhere overnight. Confluent cultures were scratched using a sterile 10 μL pipette tip and rinsed with PBS. Cells were pretreated with L6H21 at 10 μM, MD2 neutralizing antibody at 1 μg/mL, or DMSO vehicle in the presence of mitomycin C (8 μg/mL) in calcium-free RPMI-1640 medium for 1 h. Mitomyocin C was utilized to inhibit cell proliferation. Cells were then challenged with 1 μg/mL LPS, and photomicrographs of cells migrating across the scratched line were recorded at 0, 24 and 48 h. Migration was quantified by determining the distance covered from the scratched line to the middle, relative to the control.

### Western blot analysis

Lysates were prepared from tumor specimens or cells following indicated treatments. Cytosolic and nuclear protein was extracted according to a manufacture's instruction (Beyotime Biotechnology, Shanghai, China). Protein concentration was measured by the Bradford assay (Bio-Rad Laboratories, Hercules, CA). Protein aliquots were separated by 10-12% SDS-PAGE, transferred to nitrocellulose membranes (Bio-Rad Laboratories), and blocked with 5% non-fat milk in TBST at room temperature for 1.5 h. The membranes were incubated with primary antibody at 4°C overnight, followed by HP-conjugated secondary antibodies for 1 h. Immunoreactive bands were visualized using enhanced chemiluminescence reagents (Bio-Rad Laboratories), and densitometric analysis was made using ImageJ version 1.38e (National Institutes of Health, Bethesda, MD). Values were reported as normalized to their respective controls.

### Real-time quantitative PCR

RNA from tissues and cells was isolated using TRIZOL (Invitrogen). Both reverse transcription and quantitative PCR were carried out using a two-step M-MLV Platinum SYBR Green qPCR SuperMixUDG kit (Invitrogen, Carlsbad, CA). Eppendorf Mastercycler ep realplex detection system (Eppendorf, Hamburg, Germany) was used for q-PCR. The primers for IL-6, TNF-α, TGF-β, ICAM-1, VCAM-1 and β-actin were obtained from Invitrogen, and are shown in Supplementary file (Table S1). Transcript levels were normalized to β-actin.

### Cell transfection for MD2 gene silencing or overexpression

CT26.WT cells were transfected with siRNA target sequences for MD2 or negative control siRNA (GenePharma, Shanghai, China) using 0.1 μM siRNA in 2 μg/mL Lipofectamine® 2000 reagent (Invitrogen) for 6 h. Media was then changed to normal growth media for use in studies. Sequences of siRNA are provided in Supplementary file (Table S2).

#### Gene overexpression

The recombinant plasmid vector MG51098-NH coding MD2 protein was obtained from Sino biological Inc, China. The MG51098-NH plasmid was transfected into colon cancer cell line (CT26.WT) for 6 h using Lipofectamine 3000 reagent (Invitrogen, Carlsbad, CA, USA) according to the manufacturer's protocol. After 18 h post transfection cells were treated with L6H21 for 1 h, and then stimulated with LPS. Finally, MD2 expression and NF-κB p65/IL-6 in CT26 cells was confirmed by Western blotting and ELISA analysis, respectively.

### Co-immunoprecipitation assay

Cell lysates were incubated with anti-TLR4 antibody at 4°C overnight, and protein A+G agarose was added to precipitate the antibody-protein complexes at 4°C for 4 h. Immunoprecipitates were then probed for MD2 by immunoblotting.

### Nuclear NF-κB (p65) translocation

Following relevant treatments, NF-κB activation was determined in cells by detection of fluorescent-labelled NF-κB p65 subunit using the translocation kit (Beyotime Biotech, Nantong, China). Cells were counterstained with the nuclear stain, DAPI, and viewed by fluorescent microscopy at 400× magnification.

### Enzyme-linked immune sorbent assay (ELISA)

After treatment, 100 µg protein samples were prepared from cells or tumor tissue. Expressions of pro-inflammatory cytokine interleukin-6 (IL-6) in cell lysate and tumor tissue were assessed using commercially available ELISA kits (eBioscience, USA).

### Histological analysis

Colon cancer tissue as well as liver, heart and kidney samples were harvested from mice from the experimental groups. Tissue samples were fixed in 4% neutral-buffered formalin for 48 h, dehydrated, cleaned in xylene, and embedded in paraffin. Samples were sectioned at 5-micron thick sections, and stained with hematoxylin and eosin (H&E) for routine histology. For immunohistochemistry, colon tumor tissue sections were deparaffinized, hydrated and subjected to heat-induced antigen retrieval. Sections were incubated with relevant primary antibodies overnight at 4 °C, followed by incubation with secondary antibodies 2 h at room temperature. Diaminobenzidine (DAB) was used for detection of the antigenic sites. The sections were counterstained with hematoxylin for 30 seconds, mounted and viewed under a light microscope. DAB positivity was measured by Image J software (20× magnification, Nikon, Japan).

### Statistical analysis

Data were represented as mean ± SEM of three or four independent experiments. We used one-way ANOVA followed by Dunnett's post hoc test when comparing more than two groups of data and one-way ANOVA, non-parametric Kruskal-Wallis test, followed by Dunn's post hoc test when comparing multiple independent groups. Survival data were presented as Kaplan-Meier survival curves and differences between groups were analyzed by the log-rank test using GraphPad Prism 5.0 (San Diego, CA). A *P* value < 0.05 was considered to be statistically significant. Post-tests were run only if F achieved *P* < 0.05 and there was no significant variance in homogeneity.

## Results

### MD2 expression is increased in human colon cancer

**Figure 1:**
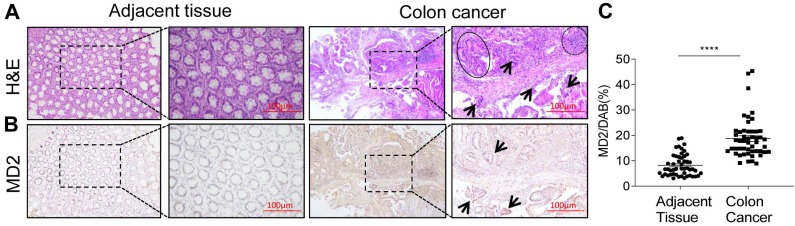
** MD2 expression is upregulated in human colon cancer**. Human colon biopsy samples were prepared by immunohistochemical method for analysis; n=50. (A) Representative image stained by hematoxylin and eosin (H&E) showing normal adjacent colon tissue (left 2 panels) and colon cancer tissues (right 2 panels); boxed area indicates magnified area. (B) Representative images of immunochemical detection for MD2 (brown); normal adjacent colon tissue (left 2 panels) and colon cancer tissues (right 2 panels); boxed area indicates magnified area; scale bar = 100 µm. (C) Quantification of MD2 immunoreactivity of colon tissues as determined by Image J from colon biopsy samples. Data are shown as mean±SEM, n=50, ****P<0.0001 compared to normal colon tissues.

We first examined the expression levels of MD2 protein of a panel of 50 human colon cancer specimens and their adjacent non-neoplastic tissues. MD2 expression was detected by immunohistochemical method using anti-MD2 antibody. Representative H&E image showing normal tissue morphology (Fig. 1A, left panels). Colon cancer growth shows multiple aberrant crypt foci lined with pleomorphic hyperchromatic nuclei (circle), densely packed inflammatory cell infiltrations (arrow mark) and prominent lymphoid aggregates (dotted circle) (Fig. 1A, right panels). Immuno-histochemcal detection of MD2 in adjacent non-neoplastic tissues (Fig. 1B, left panels), and in colon cancer tissues shows dark brown development in abnormal crypts (arrow mark) (Fig. 1B, right panels). Quantification of MD2 staining intensity indicated increased levels in colon cancer specimens compared to control tissues (Figure 1C). The data indicated that increased MD2 expression was linked to colon cancer growth, implicating MD2 in contributing to the pathogenesis of colon cancer.

### MD2 regulates colon cancer cell migration and invasion

**Figure 2:**
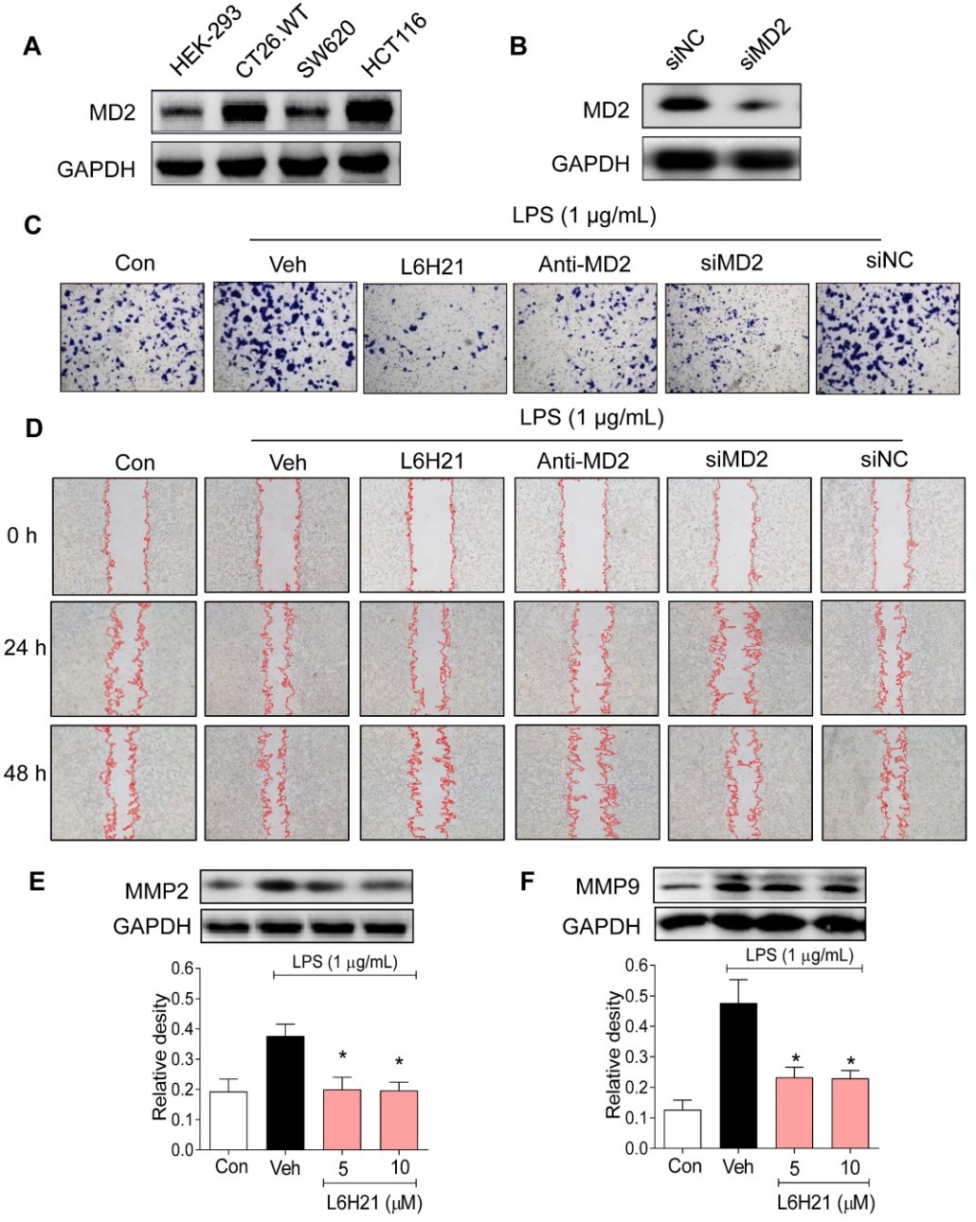
** MD2 blockade inhibits colon cancer cell motility and invasiveness.** (A) Western blot analysis of MD2 in colon cancer cell lines (mouse CT26.WT and human SW260 and HCT116) and in non-cancer cells human embryonic kidney cells (HEK-293); GAPDH was used as loading control. Representative images are shown; n=3. (B) Western blot showing MD2 knockdown (in duplicates) by transfection of siRNA target sequences (siMD2) or negative control sequences (siNC) in CT26.WT cells; GAPDH as loading control. Representative images are shown; n=3. (C) Representative images of CT26.WT cells stained with crystal violet (blue) that have migrated across Matrigel-coated transwell filters; MD2 blockade was made by L6H21 pretreatment (10 µM for 1 h), pretreatment with MD2 neutralizing antibody (anti-MD2; 1 µg/ml for 1 h), or transfection with siRNA target sequences (siMD2) or negative control sequences (siNC); cells were stimulated with LPS for 24 h for invasion analysis; Con=Control, Veh=vehicle control; n=3. (D) Representative image of LPS-stimulated motility of CT26.WT cells across scratched wound at 24 h and 48 h; MD2 blockade as described in (C); Con=Control, Veh=vehicle control; n=3. Quantification of data in C and D are provided in supplementary file. (E-F) Western blot analysis of matrix metalloproteinases 2 (MMP-2) 2 and 9 (MMP-9) in LPS-stimulated (30 min) CT26.WT cells pretreated with L6H21 at 5 or 10 µM for 1 h; GAPDH was used as loading control; Con=Control, Veh=vehicle control. Lower panels show densitometric quantification of immunoblots [data are shown as mean±SEM, n=3; *, *P*<0.05 compared to LPS alone].

We next investigated the potential pathogenic mechanism of MD2 in colon cancer growth using culture cells. We measured MD2 protein expression in mouse (CT26.WT) as well as human (SW620 and HCT116) colon cancer cell lines. Human embryonic kidney 293 (HEK-293) cells were used as normal control. Western blot analysis indicated that both CT26.WT and HCT116 cancer cells expressed elevated levels of MD2 compared to that of HEK293, and SW620 (Figure 2A). Two different approaches were used to create MD2 deficits in colon cancer cells as follows: i) MD2 knockdown using siRNA target sequences (Figure 2B), and ii) neutralizing antibody to MD2. Control and MD2-deficient cells (CT26.WT, HCT116, SW620) were stimulated with lipopolysaccharide (LPS; 1 µg/mL for 48 h) for assessment of cell viability. Our results showed that MD2 neutralizing antibody, or MD2 knockdown did not alter cell viability of the 3 cell lines, with exposure to LPS (Supplementary Figure S1A).

Furthermore, the effects of MD2 deficiency on invasion and migratory capacity were investigated in CT26.WT cells. CT26.WT cells were pretreated with L6H21, a MD2 inhibitor 13, or MD2 neutralizing antibody for 1 h or transfected with MD2 siRNA, and evaluated for LPS-stimulated invasion using Matrigel-coated transwell assay. Our results indicated that MD2 inhibition by each of the 3 approaches significantly reduced the ability of CT26.WT cells to invade through the Matrigel-coated filter compared to vehicle-treated or negative control siRNA groups (Figure 2C, Supplementary Figure S1B). Similarly, MD2 inhibition by each of the 3 approaches impaired the migratory ability of LPS-stimulated CT26.WT and HCT116 cells as demonstrated by the *in vitro* model of scratch/wound healing assay (Figure 2D, Supplementary Figure S1C-D, Supplementary Figure S2). The pharmacological inhibition by L6H21 was as effective as MD2 knockdown or MD2 neutralizing antibody, providing further support that L6H21 was selective for MD2. Moreover, matrix metalloproteinases (MMPs) are crucial regulators of cell migration and invasion. Western blot analysis indicated that L6H21 pretreatment in CT26.WT cells prevented the LPS-induced increases in MMP-2 and MMP-9 (Figure 2E and 2F). These data clearly support the idea that LPS-stimulated colon cancer migration and invasion were regulated by MD2.

### MD2 blockade inhibits NF-κB and inflammatory responses in colon cancer

**Figure 3:**
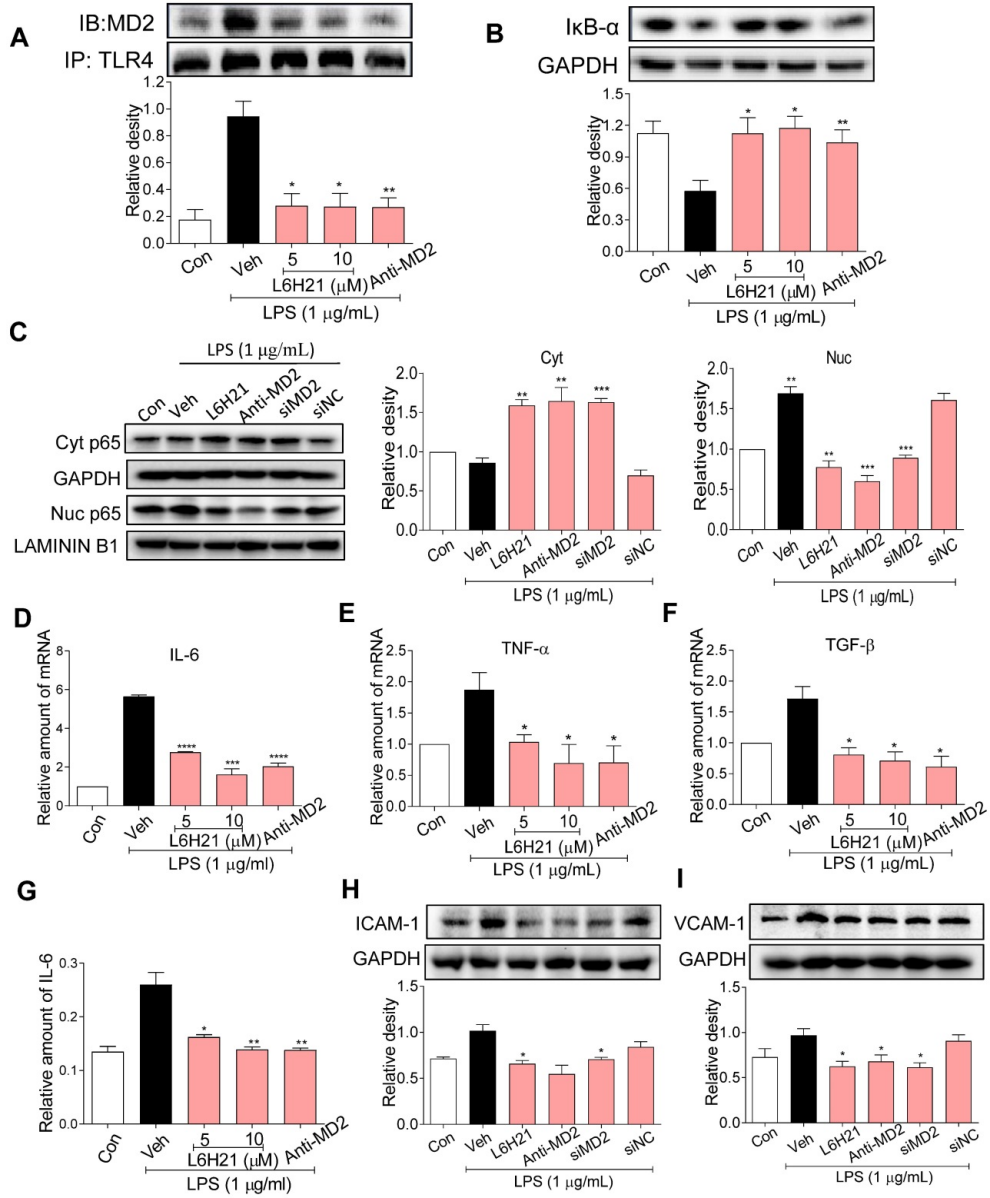
** MD2 blockade inhibits NF-κB activation in colon cancer cells**. (A) Upper panel: Representative co-immunoprecipitation analysis of interactions between MD2 and TLR4 from LPS-stimulated (15 min) CT26.WT cells pretreated for 1 h with L6H21 (5 or 10 µM) or with MD2 neutralizing antibody (anti-MD2; 1 µg/mL); IB=immunoblotting antibody, IP=precipitating antibody; Lower panel showing quantification of the immune-reactive bands and values normalized to TLR4; n=3. (B) Assessment of NF-κB activation by detecting the levels of inhibitor of IκB-α degradation in CT26.WT cells treated as described in (A); GAPDH as loading control; n=3 Lower panel: Densitometric quantification of bands; values are normalized to GAPDH. (C) Western blot analysis for nuclear translocation of p65 subunit of NF-κB in LPS-stimulated (30 min) CT26.WT cells. MD2 blockade was made by L6H21 pretreatment (10 µM for 1 h), pretreatment with MD2 neutralizing antibody (anti-MD2; 1 µg/ml for 1 h), or transfection with siRNA target sequences (siMD2) or negative control sequences (siNC). Cytosolic and nuclear protein was extracted for Western blot analysis. Representative images and quantification of the immune-reactive bands were shown, n=3. (D)IL-6, (E) TNF-α, (F) TGF-β; n=3. (G) The protein levels of IL-6 in cell lysate was detected by ELISA, values normalized to total protein; n=6. Western blot analysis (H) ICAM-1 and (I) VCAM-1 from LPS-stimulated (12 h) CT26.WT cells; treatment protocol as described in C. Lower panels: densitometric quantification of immune-reactive bands; values normalized to GAPDH, n=3. Con=Control, Veh=vehicle control. Real-time qPCR determination of ICAM-1 and VCAM-1 are provided in supplementary file. A, B, D-I: Data are shown as mean±SEM; *P<0.05, **P<0.01, ***P<0.001, ****P<0.0001 compared to LPS alone.

Since MD2 forms a complex with TLR4 and is required for TLR4 activation, we determined MD2-TLR4 complex formation by co-immunoprecipitation of LPS-stimulated CT26.WT cells. As expected, LPS increased MD2-TLR4 complex formation by >4-fold over control, but the increase was abrogated in cells pretreated with L6H21 or MD2 neutralizing antibody (Figure 3A). The LPS-induced increase in MD2-TLR4 complex formation was accompanied by 50% decreased levels of inhibitor of κB-α (IκB-α) (Figure 3B), indicating increased IκB-α degradation which corresponded with increased NF-κB activity. Significantly, MD2 blockade by L6H21 or MD2 neutralizing antibody prevented the LPS-induced degradation of IκB-α, indicating inhibition of NF-κB. To confirm these results, cytoplasmic and nuclear translocation of the NF-κB p65 subunit was evaluated using Western blot analysis. Western blot results indicated that LPS increased nuclear localization of the p65 subunit, which was inhibited by L6H21, MD2 neutralizing antibody, or MD2 knockdown in CT26.WT cells (Figure 3C). To verify these results, *in situ* immunofluorescent assay was performed and similar results were observed (Supplementary Figure S3). Next, to determine whether L6H21 efficiently inhibits MD2 and its downstream NF-κB signaling, CT26 cells were transfected with MD2 overexpression plasmid. Interestingly, our data show that overexpression of MD2 promoted TLR4/MD2 complex formation (Supplementary Figure S4A-B) and activated the downstream NF-κB/IL-6 signaling axis (Supplementary Figure S5). Also, overexpression of MD2 increased CT26.WT cell migration in a time- dependent manner (Supplementary Figure S4C-D). However, L6H21 significantly reversed these effects via suppressing TLR4/MD2 signaling pathway (Supplementary Figure S4 and S5). Collectively, these data indicate that MD2 blockade prevented the LPS-stimulated upstream MD2-TLR4 complex formation as well as the subsequent NF-κB signaling in colon cancer cells.

We have previously shown that L6H21 inhibits MD2 and attenuates LPS-induced proinflammatory cytokine production in macrophages 13. Thus, we anticipated that L6H21 would also reduce inflammatory responses in colon cancer cells as effectively as using MD2 neutralizing antibody or knockdown. For study, CT26.WT cells were pretreated with L6H21 or MD2 neutralizing antibody and stimulated with LPS (1 µg/ml for 6 h). Real-time qPCR analysis of pro-inflammatory genes indicated that LPS increased expression of IL-6, TNF-α, TGF-β, ICAM-1 and VCAM-1 by 80-120% over control, which was effectively prevented by either L6H21 or MD2 neutralizing antibody (Figure 3D-F, Supplementary Figure S6). In addition, protein levels of IL-6 (ELISA), ICAM-1 and VCAM-1 (Western blot) were significantly reduced by MD2 inhibition (Figure 3G-I). These results indicate that MD2 inhibition not only prevented the LPS-induced activation of MD2-TLR4 signaling, but also the downstream production of inflammatory molecules in colon cancer cells. Moreover, L6H21 was as effective as using MD2 neutralizing antibody or knockdown in the inhibition of LPS-stimulated responses.

### MD2 inhibitor suppresses CT26.WT lung metastasis in BALB/c mice

**Figure 4:**
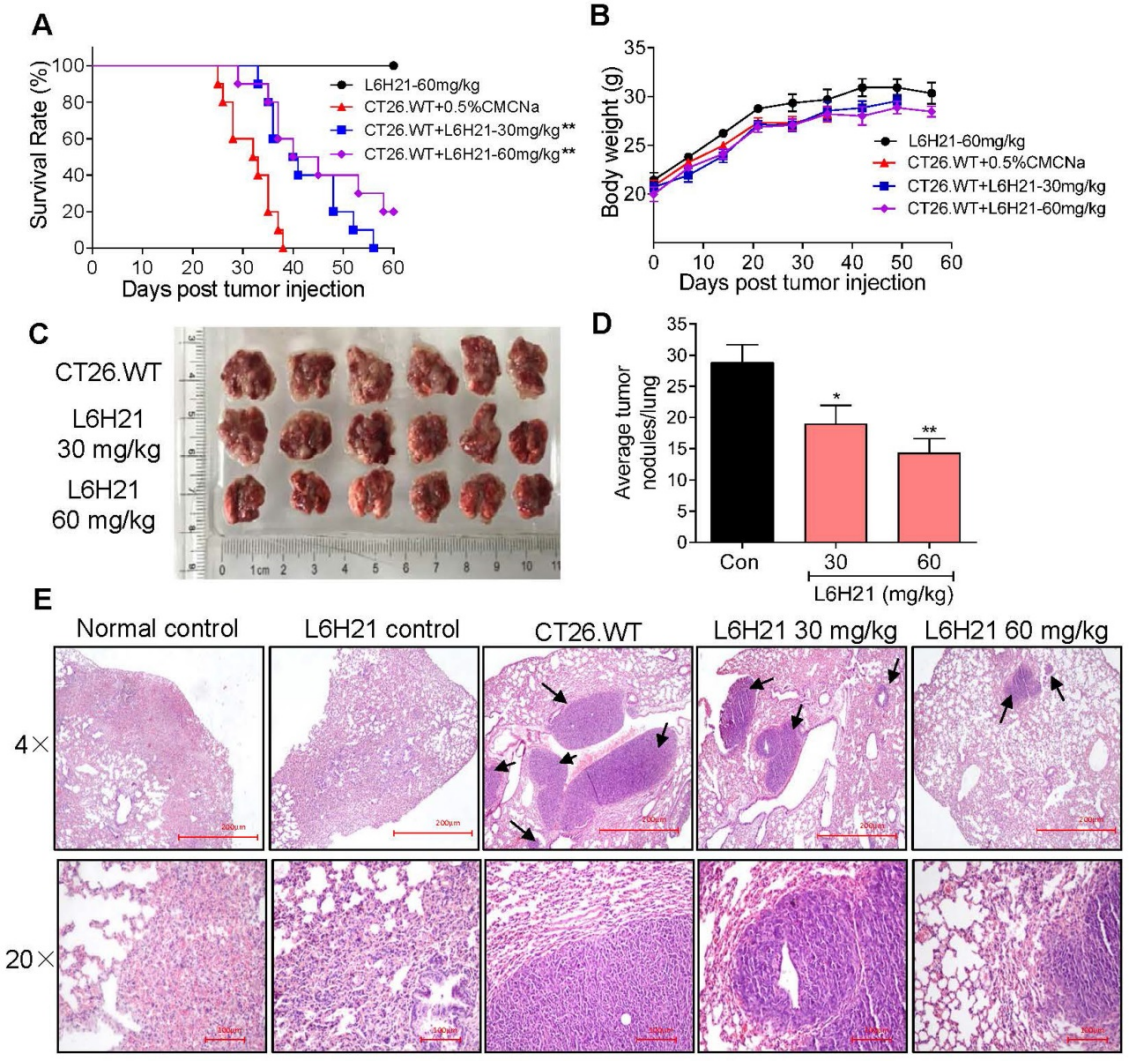
** MD2 inhibitor, L6H21, reduces colon cancer cell metastasis to lungs.** CT26.WT cells (3×10^5^ cells/mouse) were injected through tail vein of mice treated orally with L6H21 (30 mg/kg or 60 mg/kg) or vehicle (CMCNa), and lung tumor growth evaluated for up to 60 days. (A) % mouse survival post cell injection. (B) Body weight of mice post cell injection; values reported as mean±SEM, n=10. (C) Micrographs of mouse lungs 22 days post cell injection. (D) Quantification of number of tumor nodules per lung; values shown as mean±SEM, n=6, *P<0.05 and **P<0.01 compared to the CT26.WT control. (E) Lung tissue sections stained with hematoxylin and eosin; arrows indicate tumors in colon tissue (H&E); top panel: 4× magnification (micron bar= 200 µm); bottom panel: 20× magnification (micron bar=100 µm); n=6.

Our findings indicated that L6H21 was a selective and potent MD2 inhibitor in abrogating colon cancer cell activities. Therefore, we tested the inhibitory effectiveness of L6H21 in a mouse model of colon cancer cell metastasis to lungs. BALB/c mice injected intravenously with CT26.WT with or without oral administration of L6H21 (30 or 60 mg/kg), and lung tumor growth was assessed for up to 60 days. The mouse survival rate declined sharply between 25-40 days post CT26.WT injection, with no survival by 40 days (Figure 4A). The reduced survival was associated with significant lung tumor growth in CT26.WT injected mice (Supplementary Figure S7). However, the orally administered L6H21 improved survival (Figure 4A) and reduced tumor metastasis to the lungs (Supplementary Figure S7). L6H21 alone did not impair survival compared to control (Figure 4A). The body weights of mice were not different among the experimental groups (Figure 4B).

We next evaluated the effects of L6H21 on lung tumor growth during the survival period of up to 22 days post CT26.WT injection. The CT26.WT injected mice surviving up to 22 days showed enlarged tumor nodules (Figure 4C), with the number of measurable nodules estimated at 28-32 per lung (Figure 4D). Histological evaluation of these tumors indicated presence of large pleomorphic cells with prominent nucleoli, showing high proliferation index and larger nodules presenting with hemorrhage and necrosis (Figure 4E). In contrast, oral administration of mice with L6H21 (30 or 60 mg/kg) significantly inhibited lung metastatic tumor growth as evidenced by lower pathological score and decreased tumor nodules size and incidence (Figure 4C-E). Importantly, the effect appeared to be more pronounced at the dose of 60 mg/kg. These data are supportive of our *in vitro* findings that L6H21 inhibits colon cancer migration and invasion.

### MD2 blockade inhibits colitis-associated colon cancer progression

**Figure 5:**
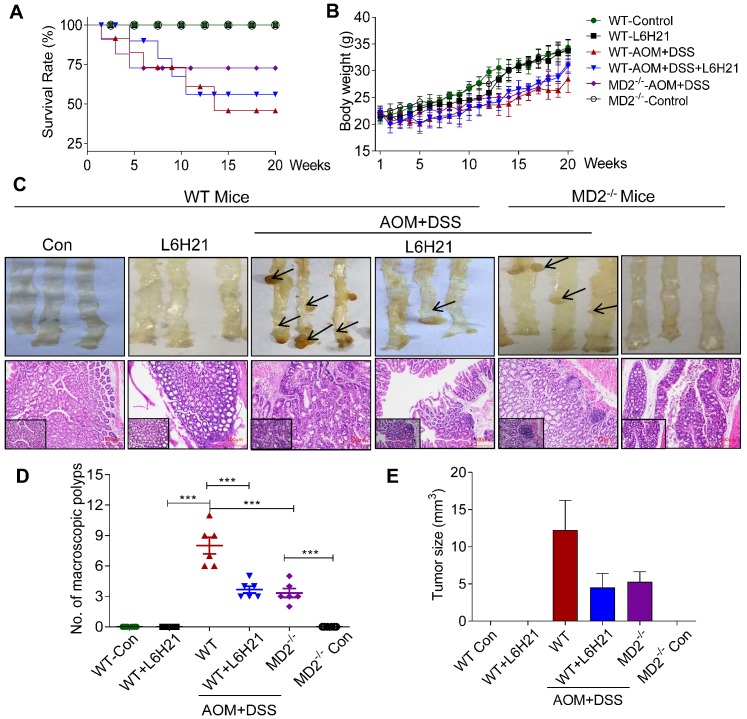
** MD2 blockade inhibits colon cancer growth in AOM/DSS mouse model.** WT or MD2^-/-^ mice were treated with AOM/DSS to produce a colitis-associated colon cancer growth. WT mice were also orally administered L6H21 (60 mg/ml) or 1% CMC-Na (vehicle control); n=10. Survival rate (A) and body weight (B) of mice was followed up to 20 days. (C) Gross images of intact mouse colons (top panel), and corresponding histological preparations stained by hematoxylin and eosin (H&E) magnification 10× (micron bar=100 µm); arrows indicate colon tumors; lower left box indicates magnified tissue area; magnification 20×. Quantification of number of tumors (D) and (E) tumor size; values are shown as mean±SEM, n=6; ***P<0.001.

The anticancer activity of L6H21was further tested using a mouse model of AOM/DSS-induced colitis-associated colon cancer, providing a particular relevant model to assess the role of MD2-linked inflammation in tumor growth. WT mice exposed to AOM/DSS showed the worst survival rate (Figure 5A) and body weight gain (Figure 5B). However, MD2 blockade improved survival of AOM-DSS-treated mice, with L6H21 showing >50% survival and MD2 knockout at around 75% (Figure 5A). Additionally, MD2 blockade improved body weight, but not to level of control mice (Figure 5B). This is indicated by the growth rate of mice (the difference between final and initial body weights, Supplementary Figure S8A).

The AOM/DSS-treated mice increased the number of macroscopic polyps (Figure 5C and 5D) and tumor size (Figure 5E). Colon length remained similar among the experimental groups (Supplementary Figure S8B). H&E staining of colon tissues indicated prominent lymphoid aggregates with dysplastic cells, multiple aberrant crypt foci, surface epithelial degeneration and inflammatory cell infiltration of neoplastic cells in the mucosa and submucosal lining in mice exposed to AOM/DSS (Figure 5C). MD2 blockade by either L6H21 or knockout reduced 50% of the colonic tumor number (Figure 5D) and size (Figure 5E) in AOM/DSS-treated mice. In addition, we found continuous mucosal leakage and blood in the feces of AOM+DSS WT mice (Supplementary Figure S8C). MD2 blockade reduced the pathological changes (Figure 5C, lower panel) and blood in the feces associated with AOM/DSS (Supplementary Figure S8C).

We also analyzed the effects of AOM/DSS and potential toxicity of L6H21 on other vital organs, i.e., heart, kidney, and liver. Our results showed that in AOM/DSS-treated mice, heart and kidney tissues appeared histologically similar to that of control mice (Supplementary Figure S9). However, liver tissue of AOM/DSS-treated mice presented with occasionally nuclear pleomorphism and inflammatory cell infiltrates in the portal triad, which might be caused by toxic metabolites of AOM (Supplementary Figure S9). L6H21 treatment did not induce pathological changes of heart, kidney, and liver tissues (Supplementary Figure S9), which was consistent with that observed for colon tissues (Figure 5C, lower panel). These findings indicated that AOM/DSS-induced inflammation was restricted mostly to the gastrointestinal tract, with some involvement of liver tissue. Also, L6H21 showed no toxicity and did not impair tissue morphology of vital organs of the body.

### MD2 blockade inhibits downstream NF-κB activation in the mouse model of AOM/DSS-induced colon cancer

**Figure 6:**
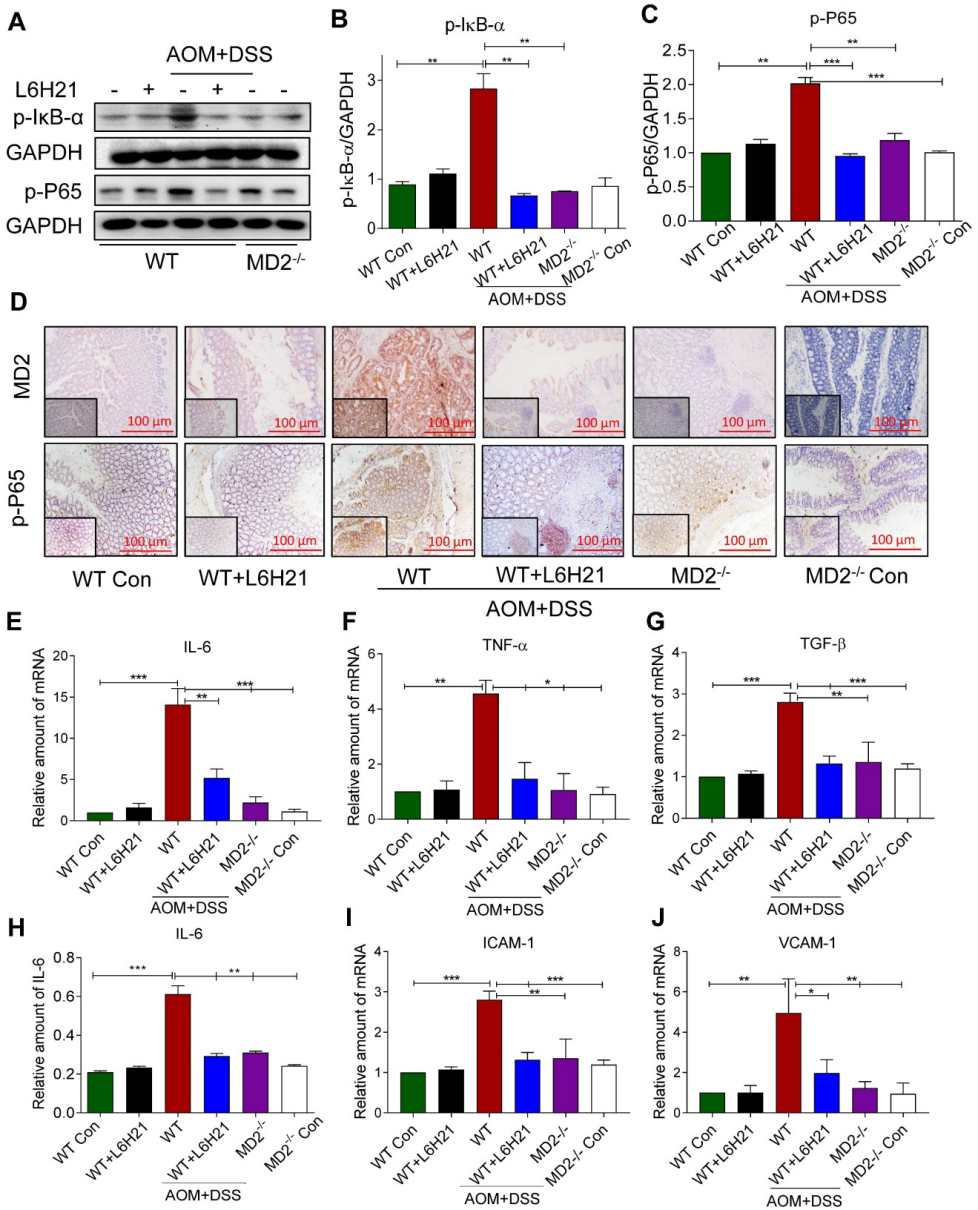
** MD2 blockade inhibits NF-κB activation in AOM/DSS mouse model.** WT or MD2^-/-^ mice were treated with AOM/DSS to produce a colitis-associated colon acncer growth (Methods). WT mice were also orally administered L6H21 (60 mg/ml) or 1% CMC-Na (vehicle control); n=10. (A) Western blot analysis of phosphorylated IκB-α and phosphorylated NF-κB P65 subunit (p-P65) in mouse colon tissues; GAPDH used as loading control. The immune-reactive bands were quantified by densitometry using Image J analysis: (B) p-IκB-α and (C) p-P65; values normalized to GAPDH. (D) Immunohistochemical staining of colon tissues from mice for MD2 (upper row; brown) and phosphorylated p65 subunit of NF-κB (bottom row; brown); n=6. Quantification (data in D) of MD2 intensity and phosphorylated NF-κB p65 subunit are provided in supplementary file. Real-time qPCR determination of pro-inflammatory genes in colonic tissue; mRNA values normalized to β-actin and reported relative to WT Con: (E) IL-6, (F) TNF-α, (G) TGF-β; n=3. (H) ELISA detection of IL-6 in colon tissue lysates; values normalized to total protein; n=6. Real-time qPCR determination of adhesion molecules in colonic tissue; mRNA values normalized to β-actin and reported relative to WT Con: (I) ICAM-1, (J) VCAM-1; n=3. Data in B, C, E, F, G, H, I, J are shown as mean±SEM; *P<0.05, **P<0.01, ***P<0.001.

We determined whether the protective mechanism of MD2 blockade was linked to inhibition of NF-κB activity in the mouse of AOM/DSS-induced colon cancer. Lysates prepared from colonic tumors of AOM/DSS-treated mice showed increased phosphorylated IκB-α (Figure 6A and 6B). This was accompanied by increased around 2-fold increase in phosphorylated NF-κB p65 subunit relative to controls (Figure 6A and 6C). The findings indicated that NF-κB activity was significantly elevated in colon tissue of AOM/DSS-treated mice. MD2 blockade with L6H21 or knockout prevented the increases in phosphorylated IκB-α and NF-κB p65 subunit in the colonic tissue of AOM/DSS-treated mice (Figure 6A-C). Immunostaining of colonic tumor tissues in AOM/DSS-treated mice showed a 5-fold increase in MD2 expression relative to controls, which was inhibited by L6H21 or MD2 knockout (Figure 6D, top row; Supplementary Figure S10A). Additionally, the immunostaining indicated a 4-fold increase in phosphorylated p-65 subunit in the colonic tissue of AOM/DSS-treated mice, which was prevented by MD2 blockade (Figure 6D, bottom row; Supplementary Figure S10B). We confirmed the inflammatory state of the colonic tissue in AOM/DSS-treated mice as indicated by >2-13 fold increases of IL-6, TNF-α and TGF-β mRNA expression (Figure 6E-G) and protein level of IL-6 (Figure 6H, ELISA), which was abrogated by MD2 blockade (Figure 6E-H). These latter observations indicted that the inflammatory signaling by NF-κB and the inflammatory state of colonic tissues were MD2-dependent in the AOM/DSS colitis cancer model. In addition, adhesion molecules ICAM-1 and VCAM-1 gene expression levels were significantly increased in AOM/DSS induced mice, which were significantly decreased on treatment with L6H21 or MD2 blockade (Figure 6I and 6J). Taken together, our *in vivo* studies mirror our *in vitro* findings and show that MD2 inhibitor L6H21 exhibits greater inhibitory activities on colon tumors by targeting MD2 and its downstream signaling components.

## Discussion

MD2 has been well recognized as an indispensable accessory protein linking LPS and TLR4 15, 16, and targeting MD2 could effectively attenuate LPS-induced inflammatory response and sepsis 13, 17, 18. Recently, we further prove that MD2 could also be an attractive therapeutic target for the treatment of many non-infectious chronic inflammatory diseases, including cardiac/kidney injuries induced by hypertension 19, obesity 20, 21, and diabetes 22. All these data suggest that MD2 can be a potential anti-inflammatory target in both acute and chronic diseases. Since chronic intestinal inflammation plays a key role in CRC initiation and progression 23, we evaluated the role of MD2 in TLR4 mediated-inflammatory responses and metastatic colon cancer growth *in vitro* and *in vivo*. The central findings from the current study provided strong evidence linking MD2-TLR4 to colon cancer growth: i) MD2 was upregulated in human colon cancer tissue, mouse colon cancer tissue in model of colitis-associated colon cancer, and colon cancer cell lines; ii) MD2 blockade prevented LPS-induced motility and invasiveness of colon cancer cells; iii) MD2 blockade significantly reduced colon cancer cell metastasis to lungs and growth in colitis-associated colon cancer, and iv) MD2 blockade abrogated NF-κB activation in mouse tumor tissue and LPS-stimulated colon cancer cells. Significantly, the pharmacological MD2 blockade using L6H21 was as effective in the anti-cancer activity as blockade using MD2 knockout, knockdown with siRNA, or treatment with neutralizing antibody.

So far, studies of CRC research have mostly focused on TLR4. Increased TLR4 expression correlates with advanced CRC stage and decreased survival in CRC patients 23, 24. Fukada and coworkers reported that TLR4 is also upregulated in inflammation-associated CRC in both humans and in experimental models 25. Furthermore, TLR4 signaling is related to colitis-associated neoplasia and CRC metastasis 9. These findings are further corroborated by immunohistochemical detection of increased expression of TLR4 and MD2 in colon adenomas 26. In addition, mice lacking TLR4 are protected from developing colon cancer 25. Eritoran, a TLR4 inhibitor, is reported to decrease cancer cell proliferation and increase apoptosis in mouse model of colonic carcinoma 27. Similarly, our study proved that blocking MD2 not only markedly decreased colon cancer cell metastasis to lungs, but also inhibited the growth of colitis-associated colon cancer, via blocking the TLR4/MD2 complex formation and inhibiting the following pro-inflammatory NF-κB activation. The highly effective anti-cancer activity of MD2 blockade strategies was likely attributed, at least in part, to this upregulated MD2 and TLR4 in colon cancer. Also, MD2 is soluble and can be directly secreted through the cell outer membrane with the modification of the first 16 hydrophobic amino acid secretory signal peptides 28. Soluble MD2 in plasma of patients with severe sepsis is known to promote the LPS response in TLR4-expressing epithelial cells of lungs and other organs 29, suggesting that soluble MD2 could be a pivotal mediator of organ inflammation. Therefore, targeting both the cell membrane and soluble MD2, rather than TLR4, may be more effective in further application in clinic. In summary, these findings underscore MD2 in promotion of colon cancer and provide an important target for anticancer strategy.

Our findings indicated that NF-κB was a crucial regulator in driving colon cancer growth. This is supported by observations that MD2 blockade of colon cancer growth in the mouse models, LPS-induced CT26.WT cell motility and invasiveness was associated with inhibited NF-κB activation. The family of NF-κB transcription factors is believed to be strongly linked with inflammation-induced tumor development and progression, regulating pro-inflammatory target genes such as IL-6, IL-8, and TNF-α 30. We found that colon cancer tissue in the mouse model as well as LPS-stimulated CT26.WT cells were in an inflammatory state as indicated by increased expression of TNF-α, IL-6 and adhesion molecules. Moreover, the inflammatory state of the colon cancer tissue and cells were attributed to primarily activation of the MD2-TLR4 signaling complex, since L6H21 reduced interaction between TLR4 and MD2 in the CT26.WT cells. Consistent with this role, MD2 blockade by L6H21, MD2 knockdown, and MD2 neutralizing antibody also reduced NF-κB activation and suppressed expression of proinflammatory molecules in colon cancer cells and tissues. MMPs are also target genes of NF-κB, and are key regulators of epithelial to mesenchymal transition, a phenotype acquired by cancer cells allowing for enhanced motility and invasiveness 31. We found that MD2 blockade inhibited both LPS-stimulated CT26.WT motility and invasiveness, which was associated with abrogation of the LPS-stimulated increases in MMP-2 and MMP-9 expression. Moreover, MMP activity may also be regulated by TNF-α malignant cell progression, invasion and metastasis 32.

Results obtained from cell culture studies and experimental mouse models indicated that the pharmacological inhibitor, L6H21, was as effective in anti-cancer activity as blockade using MD2 knockout, MD2 knockdown with siRNA, or treatment with MD2 neutralizing antibody. These findings indicated that L6H21 was likely highly selective in inhibition of MD2 in colon cancer tissue. L6H21 is a small molecule chalcone derivative developed by us and characterized with having potent anti-inflammatory activities 13, 14. The high MD2 selectivity is consistent with our report that the inhibition mechanism of L6H21 is attributed to direct binding onto MD2 13. The high selectivity property and as a small molecule chalcone derivative are advantageous features as therapeutic candidates for CRC and other gastrointestinal malignancies.

Data obtained from the AOM/DSS mouse model of colitis-associated colon cancer provide important evidence of inflammation-induced cancer growth. In this mouse model, the injected procarcinogen AOM (azoxymethane) undergoes metabolic activation in colon tissue, and oral administration of DSS (dextran-sulphate sodium salt) induces chronic colitis through disruption of the intestinal epithelial barrier, thereby exposing tissue macrophages to bacteria, and activating their innate immune system. As a result, MD2-TLR4 signalling is activated in macrophages and other cells of the gastro-intestinal tract area, leading to NF-κB activation and production of pro-inflammatory molecules. Thus, in this scenario, MD2 knockout or L6H21 administration protected against colon cancer growth, improved survival, reduced inflammation, and inhibited NF-κB activation. Significantly, this anti-cancer activity using MD2 blockade was correlated with inhibition of inflammation in colon tissue.

## Conclusions

In summary, our data show that MD2 blockade inhibited colon cancer cell migration and protected mice from inflammation-induced colon cancer development via inhibiting the activation of NF-κB pathway and reducing expression of pro-inflammatory molecules. Especially, the high selectivity of L6H21, a small molecule chalcone derivative, in targeting MD2 offers a good potential therapeutic candidate for CRC and other gastrointestinal malignancies.

## Supplementary Material

Supplementary figures and tables.

## Abbreviations

MD2: Myeloid differentiation protein 2
TLRs: Toll like receptors
MyD88: Myeloid differentiation primary response 88
TRIF: TIR-domain-containing adapter inducing interferon-β
MMP-2: Matrix metalloproteinase 2
MMP-9: Matrix metalloproteinase 9
ICAM-1: Intercellular adhesion molecule 1
VCAM-1: Vascular cell adhesion molecule 1
CRC: Colorectal cancer
FBS: Fetal bovine serum
FITC: Fluorescein isothiocyanate
PI: Propidium iodide
NF-κB: Nuclear factor kappa-light-chain-enhancer of activated B cells
IκB-α: Nuclear factor kappa light polypeptide gene enhancer in B cells inhibitor α
TNF-α: Tumor necrosis factor α
TGF-β: Transforming growth factor β
