# Selective targeting of the TLR4 co-receptor, MD2, prevents colon cancer growth and lung metastasis: Erratum

**DOI:** 10.7150/ijbs.74103

**Published:** 2022-04-26

**Authors:** Vinothkumar Rajamanickam, Tao Yan, Shanmei Xu, Junguo Hui, Xiaohong Xu, Luqing Ren, Zhoudi Liu, Guang Liang, Ouchen Wang, Yi Wang

**Affiliations:** 1 Chemical Biology Research Center, School of Pharmaceutical Sciences, Wenzhou Medical University, Wenzhou, Zhejiang, 325035, P. R. China.; 2 Department of Surgery, the First Affiliated Hospital of Wenzhou Medical University, Wenzhou, Zhejiang, 325035, P. R. China.

The authors regret that the original version of their manuscript unfortunately contained some typo and incorrect images. The statistical significance for Nuc p65 between the control and LPS vehicle groups was incorrect in Figure 3C. ** should represent *P*<0.01 vs the LPS vehicle group, but not the LPS vehicle group vs the Con. The histogram images of mRNA expressions of TGF-β and ICAM-1 in Fig.6G and Fig.6I have been misused during figure assembly. The correct versions of Figure 3C, Figure 6G and Figure 6I are listed below.

The authors confirm that the corrections made in this erratum do not affect the original conclusions. The authors apologize for any inconvenience that the errors may have caused.

## Figures and Tables

**Figure 3:**
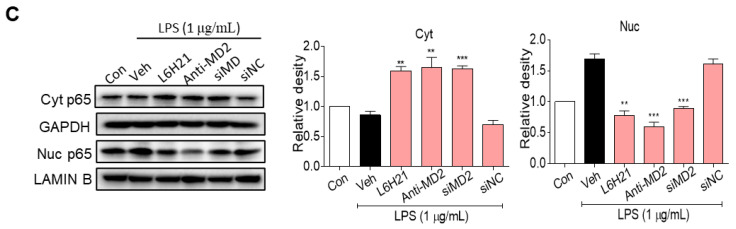
(C) Western blot analysis for nuclear translocation of p65 subunit of NF-κB in LPS-stimulated (30 min) CT26.WT cells. MD2 blockade was made by L6H21 pretreatment (10 μM for 1 h), pretreatment with MD2 neutralizing antibody (anti-MD2; 1 μg/ml for 1 h), or transfection with siRNA target sequences (siMD2) or negative control sequences (siNC). Cytosolic and nuclear protein was extracted for Western blot analysis. Representative images and quantification of the immune-reactive bands were shown, n=3. Data are shown as mean ± SEM; **, *P*<0.01, ***, *P*<0.001, compared to LPS alone.

**Figure 6:**
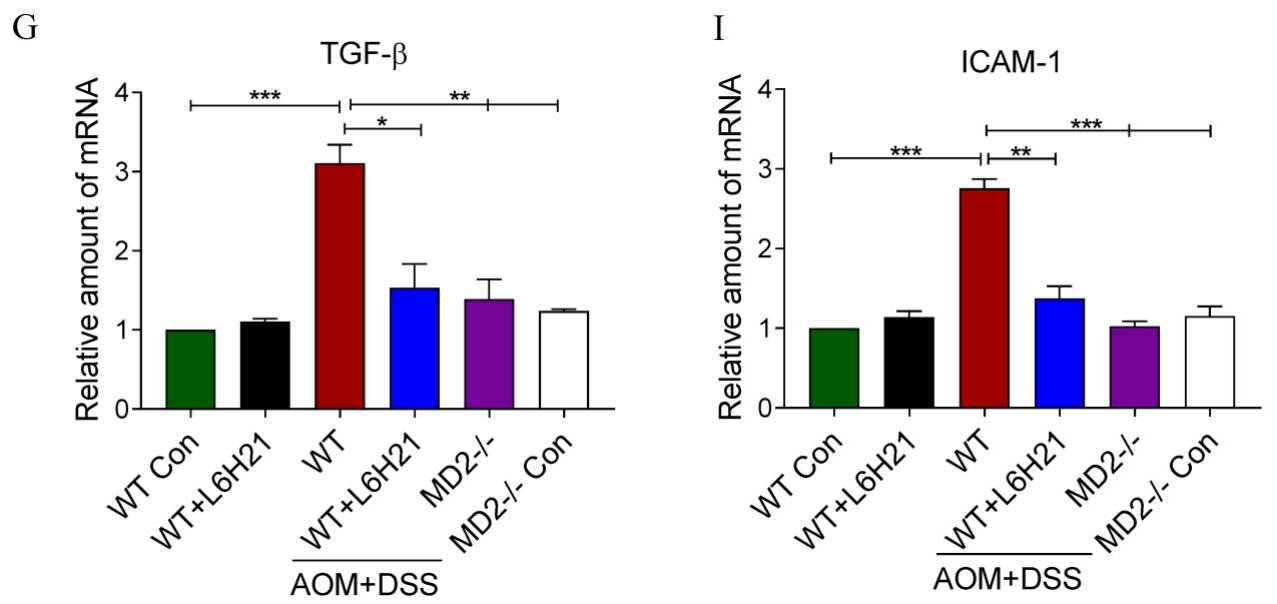
Real-time qPCR determination of pro-inflammatory gene TGF-β (G) and adhesion molecule ICAM-1 (I) in colonic tissue; mRNA values normalized to β-actin and reported relative to WT control. Data are shown as mean ± SEM; *P<0.05, **P<0.01, ***P<0.001

