# Supplementary material for: Selective targeting of the TLR4 co-receptor, MD2, prevents colon cancer growth and lung metastasis
Source: Int J Biol Sci. 2020 Feb 17;16(8):1288–301. doi: 10.7150/ijbs.39098 (PMC7085228; doi:10.7150/ijbs.39098)
Supplement: Supplementary file 1 — Supplementary figures and tables. [file ijbsv16p1288s1.pdf]

Supplementary Information

Selective targeting of the TLR4 co-receptor, MD2, prevents colon cancer growth and lung metastasis

Vinothkumar Rajamanickam[1,#], Shanmei Xu[1,#], Tao Yan[1], Junguo Hui[1], Xiaohong Xu[1], Luqing Ren[1], Zhoudi Liu[1], Guang Liang[1], Ouchen Wang[2], Yi Wang[1,*]

[1] Chemical Biology Research Center, School of Pharmaceutical Sciences, Wenzhou Medical University, Wenzhou, Zhejiang, 325035, P. R. China.

[2] Department of Surgery, the First Affiliated Hospital of Wenzhou Medical University, Wenzhou, Zhejiang, 325035, P. R. China.

[#] These authors contributed equally to this study.

Online Supplement:

1. Supplementary Table S1 and S2
2. Supplementary Figure S1-S10

Supplementary Tables

Supplementary Table S1. Primer sequences used for RT-qPCR analysis

Gene	Species	Sequence
IL-6	Mouse	5'- AAGTCCGGAGAGGAGACTTC-3'
		5'- TGGATGGTCTTGGTCCTTAG-3'
TNF-α	Mouse	5'- TGATCCGCGACGTGGAA-3'
		5'- ACCGCCTGGAGTTCTGGAA-3'
TGF-β	Mouse	5'- TGACGTCACTGGAGTTGTACGG-3'
		5'- GGTTCATGTCATGGATGGTGC-3'
ICAM	Mouse	5'- GCCTTGGTAGAGGTGACTGAG-3'
		5'- GACCGGAGCTGAAAAGTTGTA-3'
VCAM	Mouse	5'- TGCCGAGCTAAATTACACATTG-3'
		5'- CCTTGTGGAGGGATGTACAGA-3'
β-actin	Mouse	5'- CCGTGAAAAGATGACCCAGA-3'
		5'- TACGACCAGAGGCATACAG-3'

Supplementary Table S2. siRNA sequences

siRNA	Species	Sequence
MD2	Mouse	5'- GCAACUCCUCCGAUGCAAUTT-3'
		5'- AUUGCAUCGGAGGAGUUGCTT-3'
MD2	Human	5'- GAAUCUUCCAAAGCGCAAATT-3'
		5'- UUUGCGCUUUGGAAGAUUCTT-3'

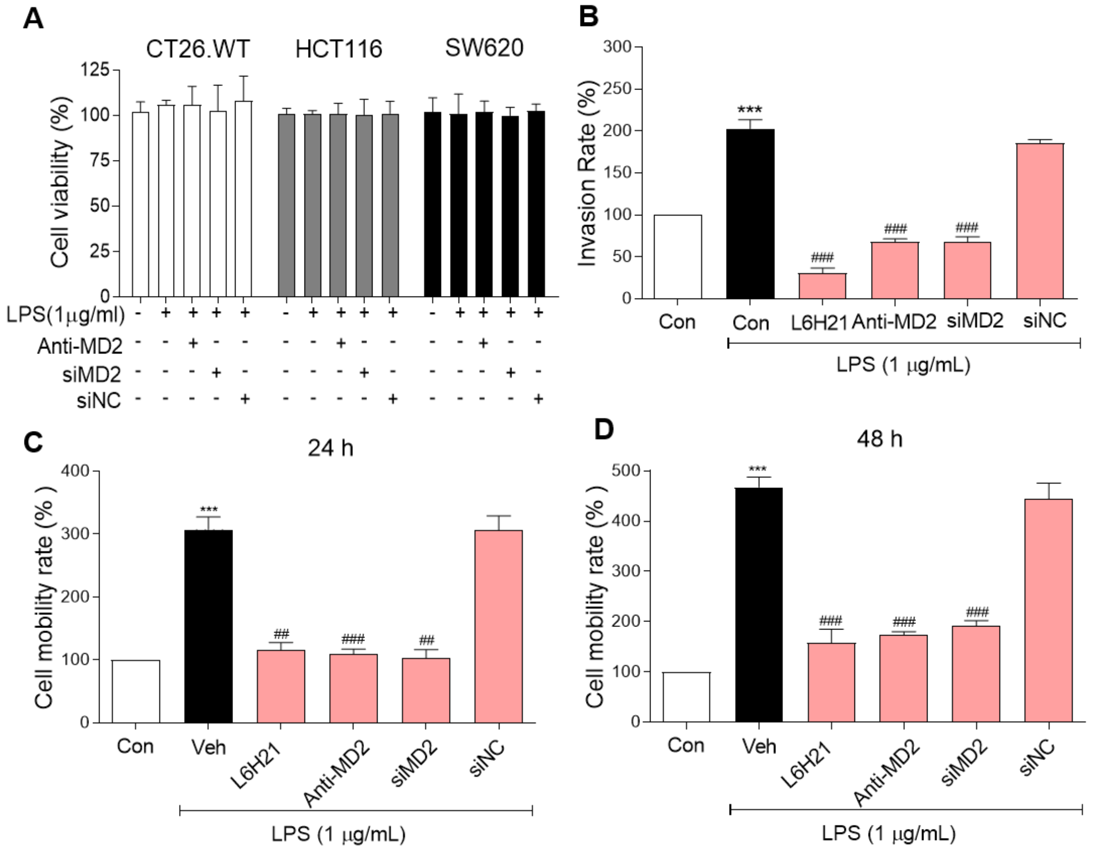

Figure S1. MD2 blockade inhibits colon cancer migration and invasion
(A) CT26.WT, HCT116 and SW620 cells were pretreated with neutralizing MD2 antibody (anti-MD2) for 1 h or transfected with siRNA target sequences (siMD2) or negative control sequences (siNC). Cells were challenged with LPS for 48 h, and cell viability was measured by MTT assay. (B) Quantification of invasion assay shown in Fig. 2C. (C-D) Quantification of scratch/wound healing assay shown in Fig. 2D. Con=Control, Veh=vehicle control; data are presented as mean±SEM, n=8. ****P*<0.001 compared to non-LPS treated control; $^{\#}$, *P*<0.05, $^{\#\#}$, *P*<0.01, $^{\#\#\#}$*P*<0.001 compared to LPS-treated control.

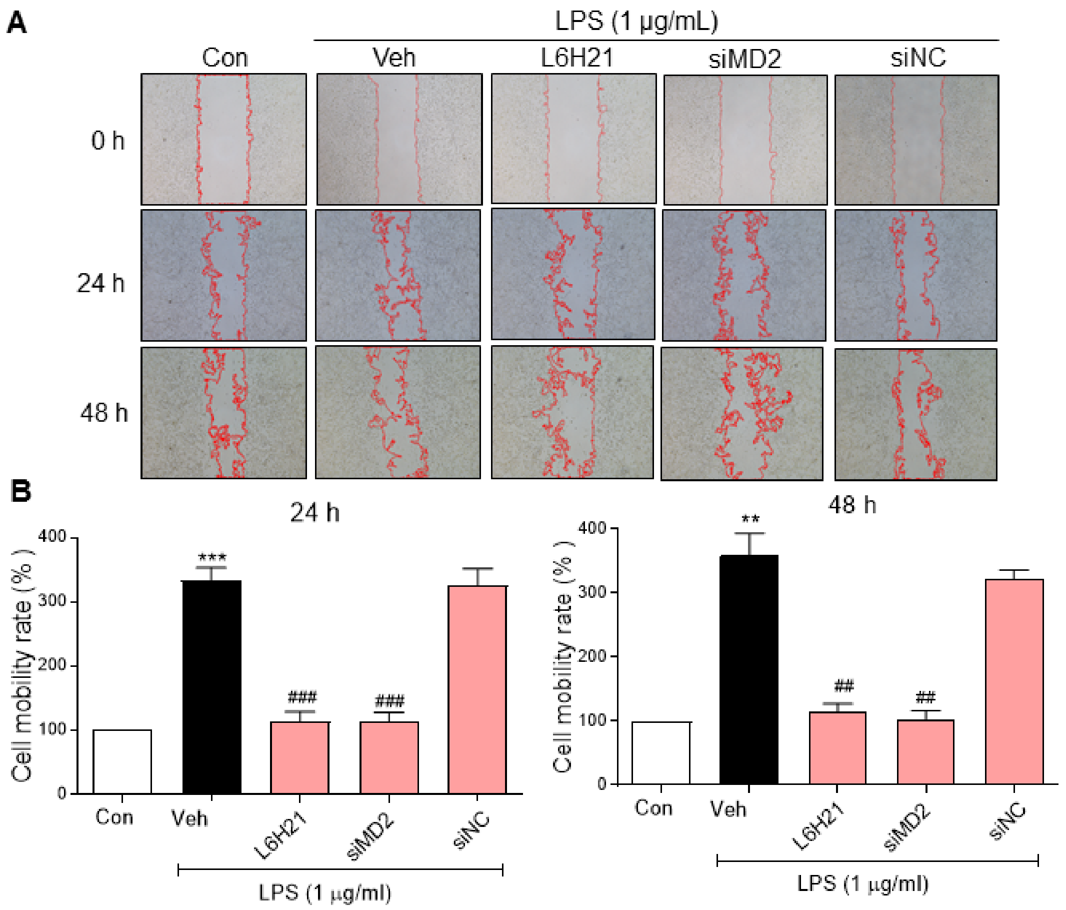

Figure S2. MD2 blockade inhibits human colon cancer cell migration
(A) Representative image of LPS-stimulated motility of HCT-116 cells across scratched wound at 24 h and 48 h. (B) Image J quantification of scratch/wound healing assay. Con=Control, Veh=vehicle control; data are presented as mean±SEM, n=3. ***P*<0.01, ****P*<0.001 compared to non-LPS treated control; $^{\#\#}$, *P*<0.01; $^{\#\#\#}$, *P*<0.001 compared to LPS-treated control.

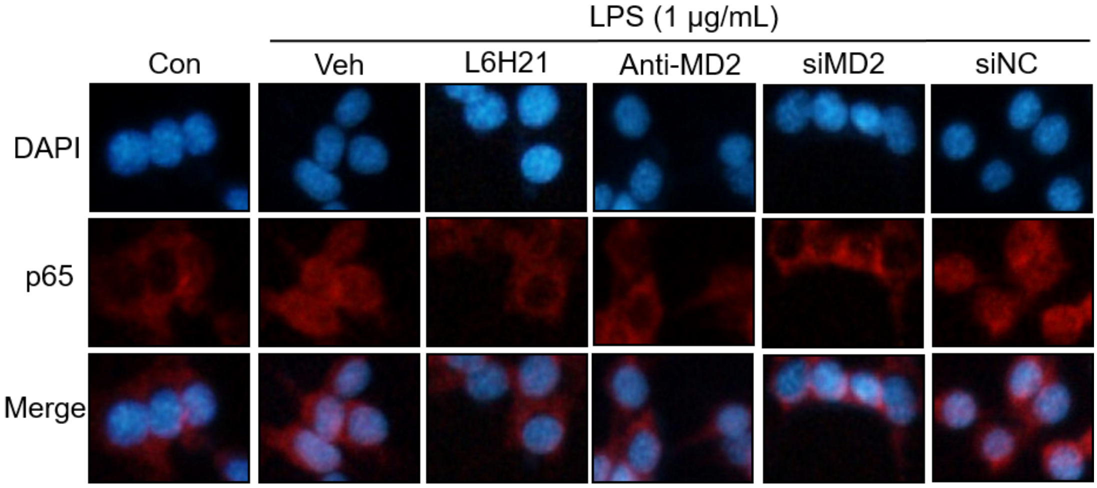

Figure S3. Immunofluorescence staining for p65 subunit of NF-κB (red) in LPS-stimulated (30 min) CT26.WT cells. MD2 blockade was made by L6H21 pretreatment (10 μM for 1 h), pretreatment with MD2 neutralizing antibody (anti-MD2; 1 μg/mL for 1 h), or transfection with siRNA target sequences (siMD2) or negative control sequences (siNC); cells were counterstained with nuclear stain, DAPI (blue); representative images were shown, n=3.

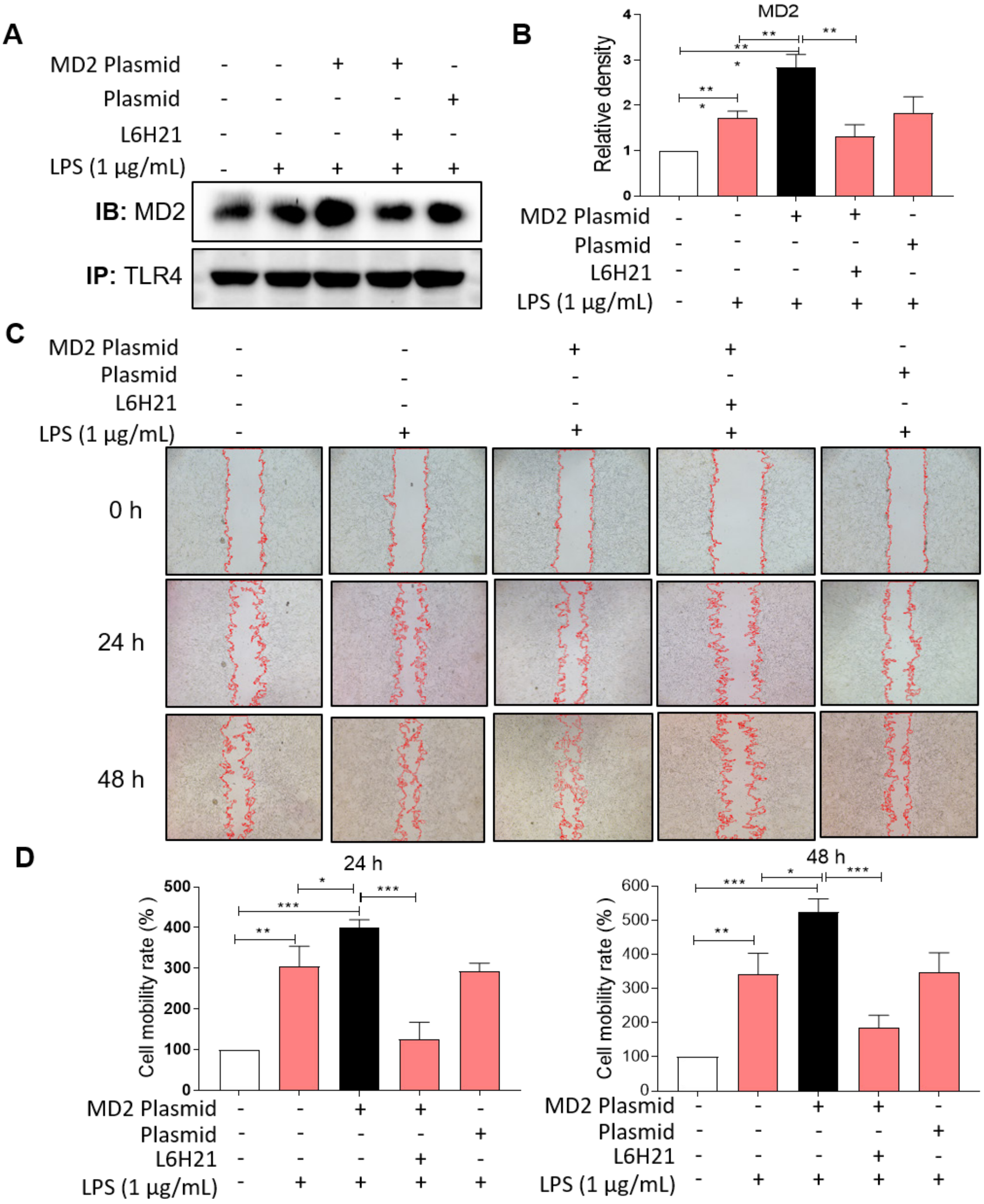

Figure S4. MD2 is involved in colon cancer migration

(A) Western blot analysis of TLR4 immuno-precipitation (IP) in colon cancer cell lines (mouse CT26.WT). MD2 plasmid=MD2 overexpression plasmid (1μg, Sino Biological. Beijing, China); plasmid=control plasmid (1μg, Sino Biological. Beijing, China); L6H21=10 μM; LPS=1 μg/mL. WT or MD2 overexpression CT26.WT cells pretreated with or without L6H21 were stimulated with LPS for 15 min. The formation of MD2/TLR4 complex was determined using IP assay. (B) Densitometric quantification of immune-reactive bands; data are presented as mean±SEM, n=3. (C) Representative images of MD2 overexpression and wide-type CT26.WT cells across scratched wound at 24 h and 48 h. (D) Image J quantification of scratch/wound healing assay. Data are shown as mean±SEM; n=3; $^{*}p<0.05$, $^{**}p<0.01$, $^{***}p<0.001$.

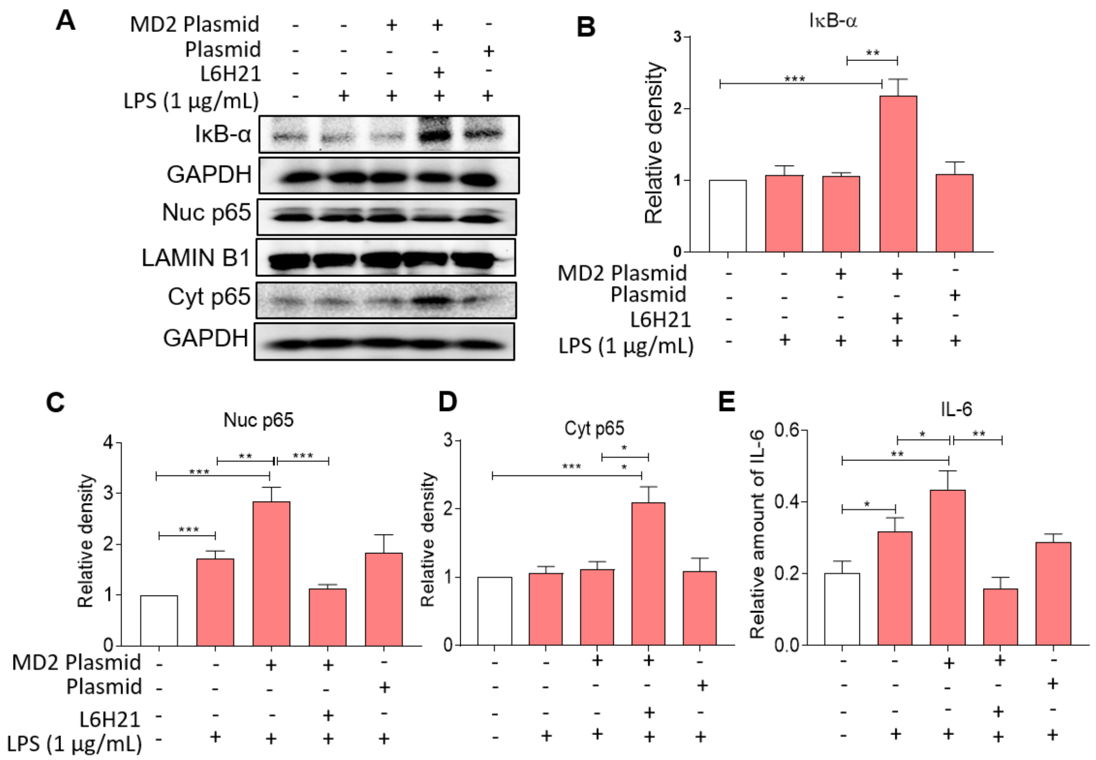

Figure S5. MD2 overexpression actives NF-κB pathway and induces inflammation in CT26.WT cells

(A) Western blot analysis of the effect of MD2 overexpression on NF-κB activation. MD2 overexpression or wide-type CT26.WT cells were pretreated with L6H21 (10 μM) for 1 h, followed by stimulation of LPS (1 μg/mL) for 30 min. The expression of I-κBα, nuclear p-65, and cytosol p65 were evaluated via Western blot assay. (B-D) Densitometric quantification of immune-reactive bands; data are presented as mean±SEM, n=3. GAPDH and Lamin B1 were used as loading control. (E) After LPS-stimulation for 6 h, the protein levels of IL-6 in cell lysate was detected by ELISA, values normalized to total protein; n=6. Data are shown as mean±SEM, n=3; $^{*}p<0.05$, $^{**}p<0.01$, $^{***}p<0.001$.

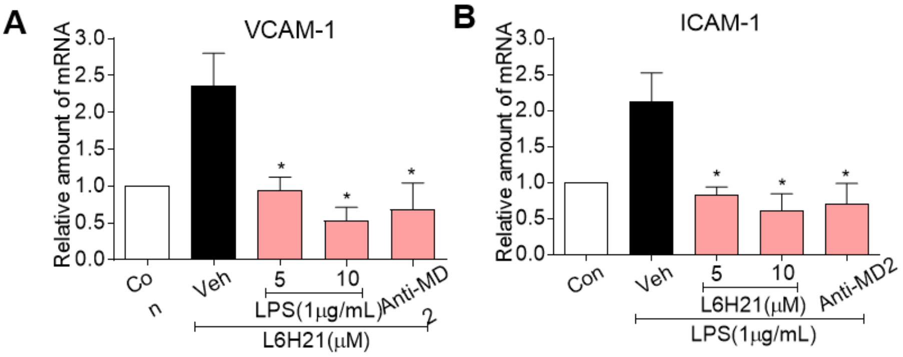

Figure S6. MD2 blockade inhibits adhesion molecule gene expression
CT26.WT cells pretreated for 1 h with L6H21 (5 or 10 μM) or with MD2 neutralizing antibody (anti-MD2; 1 μg/mL) and stimulated with LPS (1 μg/mL, 6 h). Real-time qPCR determination of adhesion molecule genes in LPS-stimulated CT26.WT cells; mRNA values normalized to β-actin and reported relative to Con: (A) VCAM-1, (B) ICAM-1; n=3. Data are shown as mean±SEM, n=3; *P<0.05 compared to LPS alone.

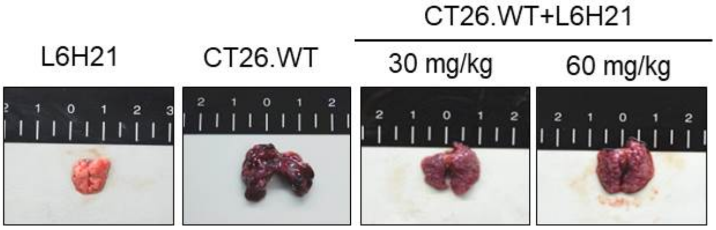

Figure S7. MD2 inhibitor suppresses colon cancer metastasis to lungs
CT26.WT cells were injected through tail vein of mice treated with L6H21, and monitored for up to 60 days. Mice were sacrificed on Day 60 post tumor cell injection. Harvested lung tissues showing metastasis in mice injected with CT26.WT cells. Representative images are shown.

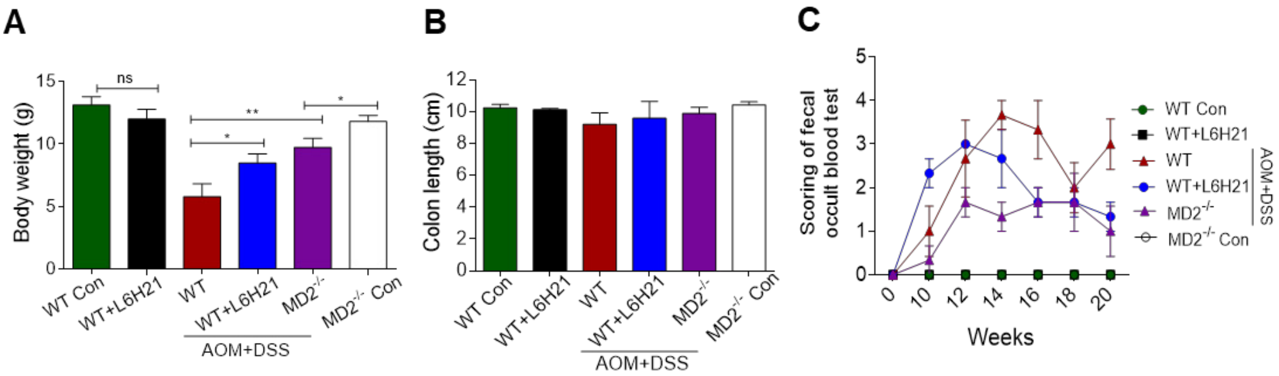

Figure S8. MD2 blockade inhibits colon cancer growth in AOM/DSS-treated mice
(A) Quantification of mouse body weight change. (B) Measurements of mouse colon length. (C) Scoring of fecal occult blood test. Blood bleeding in the stool were determined by occult blood diagnostic kit (Jiancheng Bioengineering Institute, Nanjing, China) according to the manufactures' protocol. Data are presented as mean±SEM, n=6. *, *P*<0.05; **, *P*<0.01; ns=not significant.

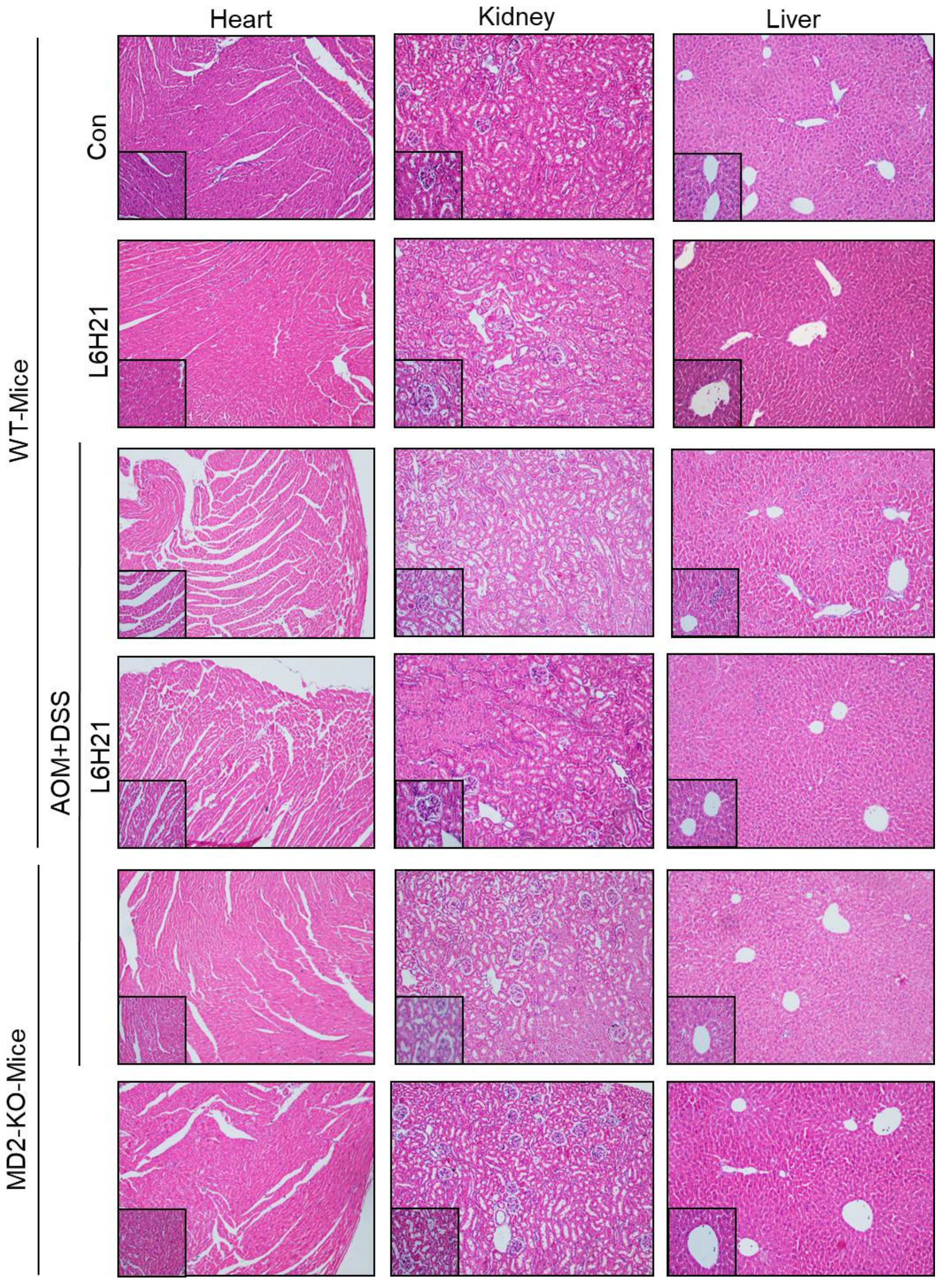

Figure S9. Effects of AOM/DSS and L6H21 on heart, kidney and liver morphology
Tissue samples were harvested from the experimental groups, and prepared by routine hematoxylin and eosin staining (H&E). Lower left box: 40× magnification; bigger box: 10× magnification. Representative images are shown.

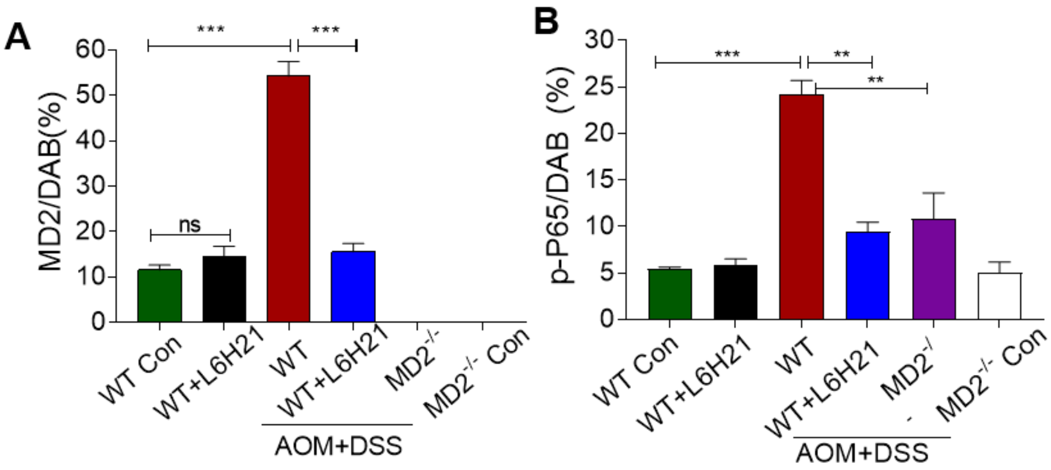

Figure S10. MD2 blockade inhibits MD2 and NF-κB activation in AOM/DSS-treated mice Image J quantification immunohistochemical staining of colon tissues from mice for MD2 and phosphorylated p65 subunit of NF-κB. Data are shown as mean±SEM, n=6 *P<0.05, **P<0.01, ***P<0.001.
